# MUS81 Participates in the Progression of Serous Ovarian Cancer Associated With Dysfunctional DNA Repair System

**DOI:** 10.3389/fonc.2019.01189

**Published:** 2019-11-15

**Authors:** Renquan Lu, Suhong Xie, Yanchun Wang, Hui Zheng, Hongqin Zhang, Minjie Deng, Weizhong Shi, Ailing Zhong, Miaomiao Chen, Meiqin Zhang, Xiaofeng Xu, Masood A. Shammas, Lin Guo

**Affiliations:** ^1^Department of Clinical Laboratory, Fudan University Shanghai Cancer Center, Shanghai, China; ^2^Department of Oncology, Shanghai Medical College, Fudan University, Shanghai, China; ^3^Department of Clinical Laboratory, Shanghai Proton and Heavy Ion Center, Shanghai, China; ^4^Department of Gynecological Oncology, Fudan University Shanghai Cancer Center, Shanghai, China; ^5^Department of Medical Oncology, Dana Farber (Harvard) Cancer Institute, Boston, MA, United States

**Keywords:** ovarian cancer, MUS81, DNA damage, homologous recombination (HR), chemotherapy

## Abstract

**Objective:** Methyl methanesulfonate ultraviolet sensitive gene clone 81 (MUS81) is a structure-specific endonuclease that plays a pivotal role in the DNA repair system of cancer cells. In this study, we aim to elucidate the potential association between the dysfunction of MUS81 and the progression of Serous Ovarian Cancer (SOC).

**Methods:** To investigate the association between MUS81 and prognosis of SOC, immunohistochemistry technology and qPCR were used to analyze the level of MUS81 expression, and transcriptional profile analysis and protein interaction screening chip were used to explore the MUS81 related signal pathways. Random amplified polymorphic DNA (RAPD) analysis, immunofluorescence and comet assays were further performed to evaluate genomic instability and DNA damage status of transduced SOC cells. Experiments both *in vitro* and *in vivo* were conducted to verify the impact of MUS81 silencing on chemotherapeutic drug sensitivity of SOC.

**Results:** The overexpression of MUS81 in SOC tissues was related to poor clinical outcomes. The transcriptional chip data showed that MUS81 was involved in multiple pathways associated with DNA repair. Deficiency of MUS81 intensified the genome instability of SOC cells, promoted the emergence of DSBs and restrained the formation of RAD51 foci in SOC cells with exposure to UV. Furthermore, downregulation of MUS81 enhanced the sensitivity to Camptothecin and Olaparib in SOC cell lines and xenograft model.

**Conclusions:** MUS81 is involved in the progression of SOC and inhibition of MUS81 could augment the susceptibility to chemotherapeutic agents. MUS81 might represent a novel molecular target for SOC chemotherapy.

## Introduction

Serous Ovarian Cancer (SOC) is a common gynecological malignancy that has increasing numbers of new diagnoses each year in China (1). Unfortunately, SOC has become an overwhelmingly lethal disease that is far from a cure. The most challenging aspect of SOC is its striking genomic instability, which is associated with abnormal DNA damage repair (DDR) pathways (2). It is now widely accepted that DDR deficiency emerged frequently in many cancer types, most notably SOC. Importantly, SOC cells have shown diverse patterns of DDR gene alterations involving cancer progression (3).

Homologous recombination (HR) is a DNA repair mechanism that promotes genomic stability through the faithful repair of double-strand breaks (DSBs) in DNA and the recovery of stalled or collapsed replication of forks (4). When genome maintenance- and replicate-involved genes are abnormal, it leads to genetic instability, as cancer cells rely on HR to resolve replicative stress before cell division. Methyl methane sulfonate ultraviolet sensitive gene clone 81 (MUS81) plays an important role in maintaining genome stability and replication fork integrity (5). Of note, recent studies have discovered that MUS81 expression levels correlate well with the evolution of various cancers such as colorectal carcinoma and lung cancer (6, 7). This study aimed to investigate the roles of MUS81 in genomic instability and SOC progression as well as to elucidate the molecular network intermediated by MUS81. DNA-damaging chemicals can have a great impact on the expression of specific genes maintaining the integrity and stability of the human genome (8). Previous evidence has indicated that MUS81 activity was required for cell survival under DNA damage, such as endogenous agents or anti-cancer compounds (9, 10). Despite the roles of this protein in coping with DNA lesions, we are still struggling to understand how they respond to the presence of DNA damage. Our previous findings revealed that MUS81 was involved in the evolution and promotion of SOC, and that deficiency of MUS81 enhanced the effects of DNA-damaging agents (11). Targeting the replication fork pathway might represent a new strategy to modulate cell response to chemotherapeutics that cause fork degradation (12). Therefore, MUS81 could be envisioned as the molecular landscape of highly replicating cancer cells and the target in sensitizing the cancer cells to anti-cancer chemicals such as arsenic (13). The roles of MUS81 could be associated with DNA repair systems and genomic instability; however, whether MUS81 contributes to cellular adaptations to genotoxic stress in SOC progression remains largely unexplored.

In this study, we performed the integrated genomic techniques to explore the amplification of MUS81 in the patients with SOC and analyzed retrospectively the relationship between MUS81 levels and clinical outcomes of SOC patients. Furthermore, the roles of MUS81 were analyzed in the progression of SOC cells using gain- and loss-of-function studies. We also used techniques such as transcriptional profile analysis and interaction protein screening arrays to explore the molecular network and DNA repair pathways regulated by MUS81, and to elucidate the molecular mechanisms of MUS81 in cell proliferation and drug sensitivity *in vitro* and *in vivo*.

## Materials and Methods

### Tissues and Cells

Specimens isolated from SOC tissues (from Tissue Bank, Fudan University Shanghai Cancer Center) were used under a protocol approved by the Ethics Committee of Shanghai Cancer Center, Fudan University (Certification No. 050432-4-1212B). Twenty-two SOC tissues (between Jan 1, 2012 and Dec 31, 2013) were selected to evaluate the association between MUS81 expression and prognosis of SOC. Clinical characteristics of these 22 SOC patients are shown in Supplementary Table 1. Tissue microarrays were constructed from another 20 patients (during 2010 and 2016, Certification No. 1603158-15) of pathologist-selected 1.0-mm tumor cores of formalin-fixed, paraffin embedded SOC specimens; each sample was in triplicate. Diagnoses were confirmed by histological analysis. Immunohistochemistry (IHC) was performed using mouse anti-MUS81 monoclonal antibody (Santa Cruz, Texas, USA) as previously described (11).

Human SOC cell lines HO8910 and SKOV3 were acquired from ATCC and cultured in RPMI 1640 medium (HyClone, Thermo Scientific, USA) with 10% fetal bovine serum (FBS, Gibco, Life technologies, USA), 100 U/mL penicillin and 100 μg/mL streptomycin (HyClone, Thermo Scientific, USA). Cells were incubated at 37°C in a humidified atmosphere with 5% CO_2_. In the study, the primary cells (named *SOC1*) were obtained from the tumor tissue of the SOC patient, described briefly as follows: fresh tissue (1 cm^3^) was resected from primary ovarian tumor and suspended in PBS to get rid of necrotic and connective tissue. The material was then gently cut into 1 mm^3^ blocks and placed in 10-cm cell culture dishes containing Medium 199 (HyClone, Thermo Scientific, USA) with 10% FBS, 100 IU/ml penicillin and 50 μg/ml streptomycin, and incubated in a humidified atmosphere containing 5% CO_2_ at 37°C. After repeated passage, a novel primary cell line *SOC1* was successfully established with more than 40 generations. Additionally, establishment of MUS81 knockdown (MUS-KD) and RAD51 knockdown (RAD51-KD) transduced cells was performed by RNAi as previously described (11). Target sequences of RNAi for *RAD51* were described in Supplementary Table 2.

### Quantitative Real-Time PCR (qPCR)

The expression levels of *MUS81, RAD51, BRCA1, BRCA2*, and *BM28 (MCM2)* were gauged by qPCR using SYBR Premix ExTaq (Takara). qPCR was performed in a 20 μL reaction containing 20 ng cDNA, 0.2 μmol/L primer and 10 μL 2 X SYBR Premix ExTaq (Takara). PCR amplification was carried out at 95°C for 5 min, then 40 cycles of 95°C for 15 s and 65°C for 40 s were conducted and analyzed on a 7500 ABI platform (Thermo Fisher Scientific) and normalized to the level of β-actin. The primer sequences of target protein are described in Supplementary Table 2.

### Comet Assay

DNA damage following UV irradiation was detected using the Comet Assay kit (Trevigen Inc.) according to the manufacture's instruction and performed as previously described (14).

### Western Blot and Flow Cytometry

Cells were lysed for total protein extraction using RIPA lysis buffer (Beyotime, P0013). Total protein was loaded and separated by SDS-PAGE and analyzed by immunoblotting for MUS81 (sc-53382, Santa Cruz, Texas, USA), BRCA1 (sc-642, Santa Cruz, Texas, USA), BRCA2 (sc-8326, Santa Cruz, Texas, USA), BM28 (#3619s, CST, MA, USA), p-H2AX (#9718s, CST, MA, USA), RAD51 (ab63801, Abcam, MA, USA), and β-actin (ab8226, Abcam, MA, USA); western blots were performed as described previously (15). Additionally, flow cytometry was performed as described previously (11).

### Random Amplified Polymorphic DNA (RAPD) Assay

The DNA was extracted from the transduced cells (1 × 10^5^) using Cell Culture DNA kit (Qiagen, 13343) according to the manufacturer's protocol and further purified with an RNase digestion. Seven arbitrary primers and PCR conditions were used as described in Ong et al. (16).

### Immunofluorescence Determination

Cells were plated onto poly-L-lysine-coated glass slides, cultured for 24 h, and then were fixed and permeabilized simultaneously at room temperature. Immunofluorescence was performed with MUS81 (Santa Cruz Biotech) and RAD51 (Abcam) staining. Alexa-Fluor 488 (Thermo Fisher Scientific, z25302), and Alexa-Fluor 546 (z25004) secondary antibodies were used. Samples were air-dried and mounted with DAPI (Thermo Scientific). Images were captured using a Zeiss LSM 700 confocal microscope (Oberkochen).

### Protein Interaction Chip

The Cell Cycle Antibody Array™ (Hypromatrix Inc., HM5000) contains 60 high-quality antibodies against a plurality of protein ligands involved in the cell cycle. The proteins captured on the array can be detected by immunoblotting. Experiments were performed according to the manufacturer's protocol.

### Docking Strategy

To elucidate potential crossing and sites of molecules interacted with MUS81, the skeletal structure of the human MUS81 binding domain was retrieved from the Protein Data Bank and the above protein array, then imported into *Discovery Studio v2.1* (Optimization for Ligand Docking, Fudan Molecular Genetic Data Center, Shanghai). The objective of molecular docking is not only to evaluate protein-ligand interactions to generate the bioactive binding structures of heterocyclic derivatives in the active site of MUS81, but also to explore the conformational space of the ligands in addition to some flexibility of active residues using a genetic algorithm.

### Apoptosis Assay

Apoptotic cells were detected by their ability to bind lactadherin, which interacts with phosphatidylserine exposed during early apoptosis, as reported previously (14).

### Drug Sensitivity Assay

For the chemosensitivity assay, single-cell suspensions were prepared and seeded in 96-well plates (5 × 10^3^ cells/well) for 24 h, then treated with 5 μmol/L Olaparib (MCE, Shanghai, China) for 12–60 h. Cell viability was assessed using a CCK-8 kit (Dojindo, Japan) according to the manufacturer's protocol. The primary cell line *SOC1* sensitivity to Camptothecin (CPT, EMD Millipore) was also performed using the CCK-8 kit. Each well was read at a wavelength of 450 nm (Synergy H4, Bio-Tek). Cell viability was calculated as follows: Viability of cells (%) = (drug group − blank) OD450/(no drug group − blank) OD450 × 100%.

The transduced SKOV3 cells' sensitivity to CPT was conducted in 6-well plates (1 × 10^3^ cells/well) in triplicate. After 24 h of cultivation, cells were treated with DMSO as the control and serial dilutions of CPT ranging from 10 to 100 nM, and then cultured for 10–14 d until visible clones appeared. Cell colonies were stained with 0.5% crystal violet and counted under a microscope for each condition. Cell viability was calculated as follows: Cell colony formation rate of cells (%) = CPT group colony number/DMSO group colony number × 100%.

### *In vivo* Experiments

The animal study was approved by the Institutional Animal Care and Use Committee of Shanghai Medical College, Fudan University (LASFDI-20140187). Female athymic BALB/c nude mice (4–6 w) were obtained from the Department of Laboratory Animal Science affiliated with Fudan University (Shanghai) and allowed to acclimatize for 1 week before any intervention was initiated. The model of SOC tumorigenesis was generated by subcutaneously injecting 5 × 10^6^ tumor cells into the right shoulders of the mice. Caliper measurements of perpendicular tumor axes were performed to monitor tumor growth, and treatment was initiated when tumors reached a diameter of approximately 6.0 mm after 2 w inoculation. The model was established by injecting CPT (dissolved in DMSO, 10 mg/kg, twice per week) into the abdominal cavity.

Image scans were performed using an Inveon micro-MRI (Siemens, Munich, Germany). Each tumor-bearing mouse was injected with 11.1 MBq (300 μCi) of ^18^F-FLT via the tail vein. Ten-minutes static scans were acquired at 1.0 h after injection, and animals were maintained under isoflurane anesthesia during the scanning period. Images were reconstructed using three-dimensional ordered-subset expectation maximization (OSEM3D)/maximum algorithm. For each microPET-CT scan, a 4.0-mm diameter spherical region of interest (ROI) was drawn over both the tumor and the contralateral muscle on decay-corrected images using Inveon Research Workplace to obtain percentage injected dose per (%ID/g) and standardized uptake values (SUV). The highest uptake point of the entire tumor was included in the ROI and no necrotic areas were allowed.

### Statistical Analysis

All statistical analyses were performed using SPSS 16.0 software. The Student *t*-test, Mann-Whitney *U*-test, and one-way ANOVA test was performed to determine significant difference statistically. Progression-free survival (PFS) was computed by the Kaplan-Meier method and analyzed with the Log-rank test. A *P-*value < 0.05 was considered significant.

## Results

### Abnormal MUS81 Expression Correlates With Malignant Features in Human Ovarian Cancer

**Figure 1:**
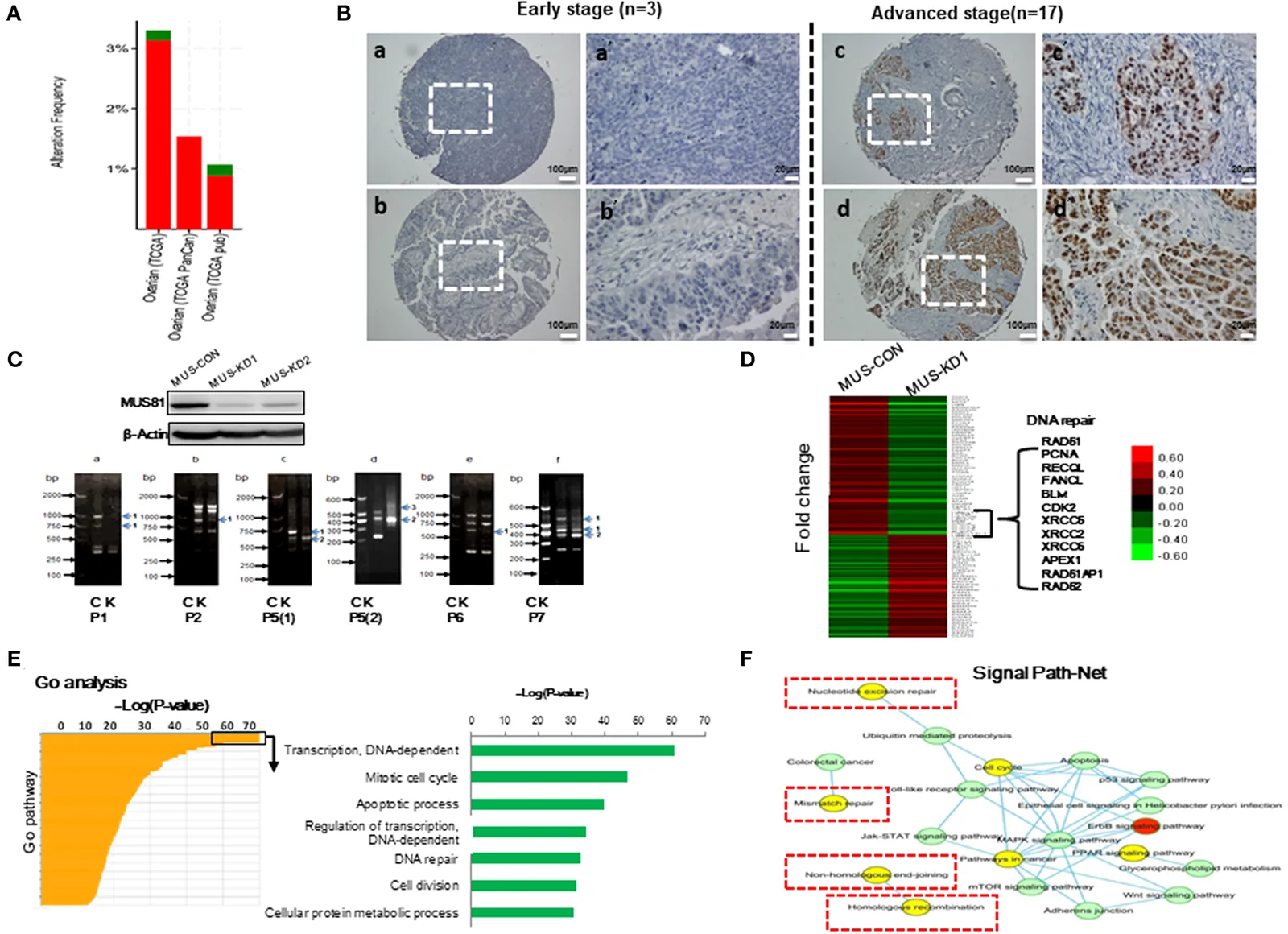
Abnormal MUS81 expression correlates with malignant features of human SOC and genomic instability of SOC cells. **(A)** MUS81 high alterations and mutations in human SOC. Data visualization was acquired from cBioportal (http://www.cbioportal.org/). **(B)** MUS81 expression is related to the progression of SOC by IHC. (a,b) Representative images of negative and weak MUS81 expression in early-stage SOC (the patient 4 and patient 6, respectively); (c,d) Representative images of moderate and strong MUS81 expression in advanced-stage SOC (the patient 8 and patient 17, respectively); (a'–d') Magnification of the white zone in (a–d). **(C)** Genomic instability is involved in the development of SOC. The level of MUS81 protein expression in transduced SKOV3 cells was determined by western blot. Changes in gene expression profiles in transduced SKOV3 cells. Band pattern changes (C: control group, K: MUS81-KD group) using RAPD analysis: (a–f) representative images of different band patterns, (a) with primer 1 (P1), (b) with primer 2 (P2), (c) with primer 5 [isoform 1, P5(1)], (d) with primer 5 (isoform 2, *P5(2)*), (e) with primer 6 (P6), (f) with primer 7 (P7); 1: band missing, 2: band intensity change, 3: band shift; **(D)** The expression profiles of MUS81 in MUS-CON and MUS-KD1 cells. **(E)** Expression profiles of different genes in the two groups were analyzed by gene ontology (GO) analysis. **(F)** Nucleotide excision repair, mismatch repair and homologous recombination (HR), etc., pathways are involved in Signal Path-Net analysis.

The level of *MUS81* expression in SOC was upregulated in our previous work (11). Similarly, there was a trend of abnormal *MUS81* expression in ovarian cancer according to TCGA (Figure 1A). To further examine the roles of MUS81 in progression of SOC, a tissue array (20 SOC patients) was performed to assess the expression of MUS81 by IHC. As shown in Figure 1B, SOC patients with early-stage presented negative or weak expression of MUS81, whereas the cases with advanced-stage presented moderate or strong expression of MUS81. Further survival analysis indicated that a higher level of MUS81 expression was associated with poorer prognosis in SOC patients (*P* = 0.029); data are shown in Supplementary Figure 1 and Supplementary Table 3. Consequently, the abnormal expression of MUS81 could be related to the progression of SOC.

### MUS81 Is Involved in Genomic Instability of SOC Cells

To explore whether MUS81 is involved in genomic instability, RAPD, a PCR-based fingerprinting technique that amplifies random DNA fragments, was used to detect genomic instability in the transduced SOC cells. As shown in Figure 1C, band profiles between MUS81-KD and control cells were significantly different, including band shifts, missing bands and band intensity changes, performed by primers 1, 2, 5, and 7, respectively. These data indicated that MUS81 could be involved in genomic instability of SOC cells.

The efficiency of DNA repair is a key factor in maintaining genomic stability (17). To explore MUS81-related signal pathways of genomic instability SOC cells, transcriptional profile was performed to analyze the differences between MUS81-KD and control cells. As shown in Figure 1D, the data revealed that the expression of multiple genes was altered in response to MUS81 silencing. Interestingly, genes involved in DNA damage repair pathways such as RAD51, PCNA, and XRCC5 were downregulated after silence of MUS81. Additionally, these results were visualized as networks by Gene Ontology (GO) analysis, and the associated pathways are grouped based on their biological roles (Figure 1E). Inhibition of MUS81 in SOC cells resulted in functional changes in some pathways, especially in the DNA-dependent transcription regulation, cell cycle progression and DNA repair system pathways (Figure 1F).

### Silencing MUS81 Enhances Dysfunction of the DNA Repair System

**Figure 2:**
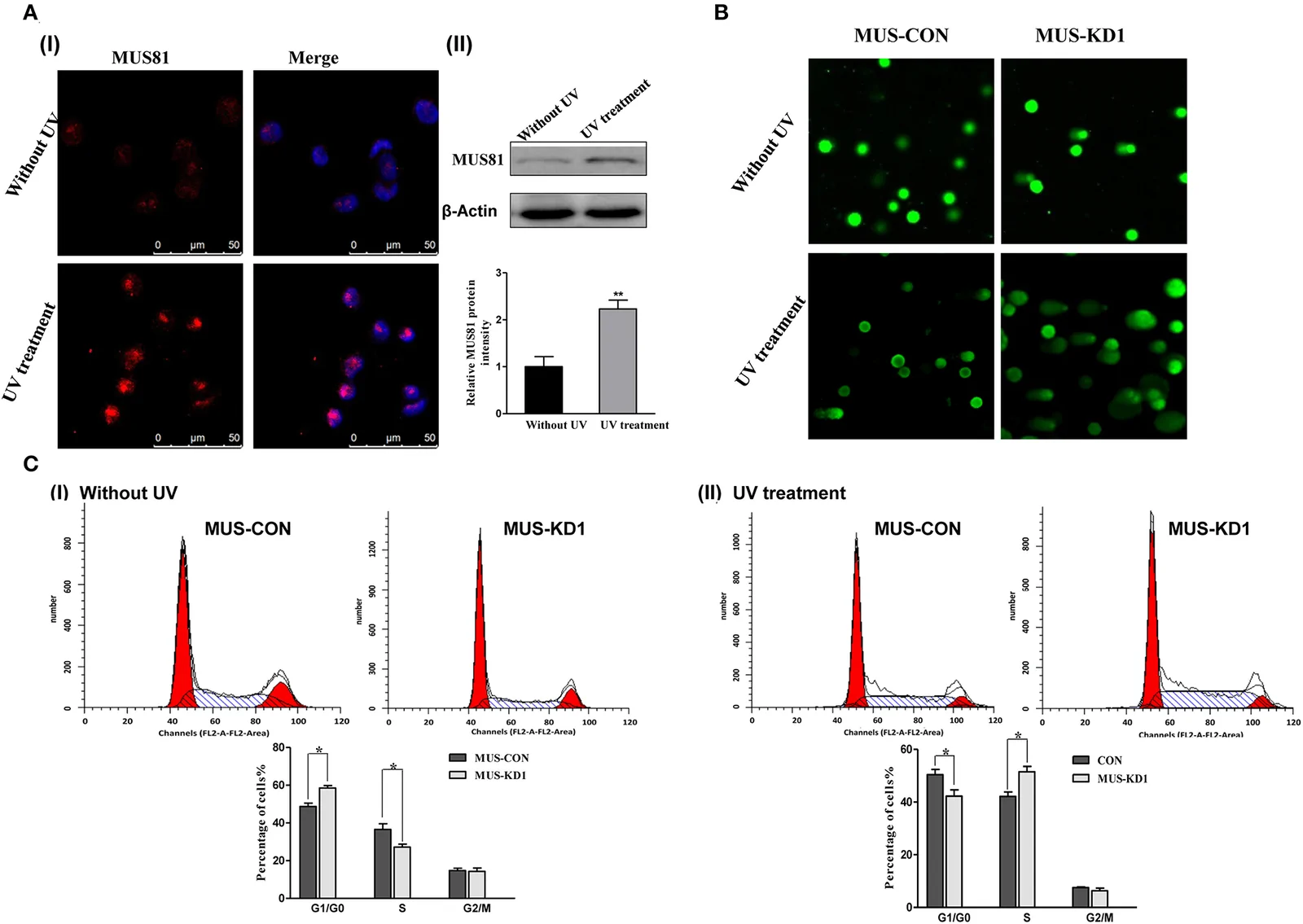
Silencing MUS81 involved in the dysfunction of the DNA repair system. **(A)** MUS81 is involved in the DNA repair system. MUS81 was upregulated after UV exposure (20 J/m^2^, recover for 24 h) using immunofluorescence (I) and western blot (II) assay. **(B)** Silencing MUS81 restrains the DNA repair system according to the comet assay. The Comet tails in MUS-KD cells were still presented after 24 h with UV treatment (20 J/m^2^). **(C)** MUS81 knockdown affected cell cycle distribution. Cell cycle distribution was assessed by flow cytometry. The MUS-CON and MUS-KD cells were exposed to UV (20 J/m^2^) and allowed to recover for 6 h. Data are presented as the mean ± SD of three independent experiments (^*^P < 0.05; ^**^P < 0.01).

The above GO-analysis results indicated that MUS81 plays an important role in the DNA repair system. Hence, we further investigated the changes of MUS81 expression in SOC cellss after UV treatment. The expression of MUS81 was increased significantly after UV exposure (Figure 2A). This suggested that MUS81 upregulation could be a transient stress response to the facilitated repair of DNA damage. A direct role for MUS81 in the DNA damage response was examined using the single-cell comet assay. As indicated in Figure 2B, the Comet tails in MUS-KD cells were still presented, whereas the majority of the control cells remained in the head, indicating that inhibition of MUS81 restricted DNA repair. Additionally, MUS81 knockdown altered the cell cycle distribution; compared with the MUS-CON group, the percentage of S phase in MUS-KD cells was significantly decreased, and cell cycle progression was arrested at G0/G1 phase (Figure 2CI). But after UV treatment, the results were dramatically changed, the percentage of G0/G1 in MUS-KD cells was significantly decreased, and cell cycle progression was arrested at S phase (Figure 2CII). In our related study, we also found that the total endonuclease activity of MUS-KD cells was decreased due to the arrest of DNA synthesis in mitosis after MUS81 silencing (18).

### Identifying Interactions Between MUS81 and Cell Cycle-Related Proteins

**Figure 3:**
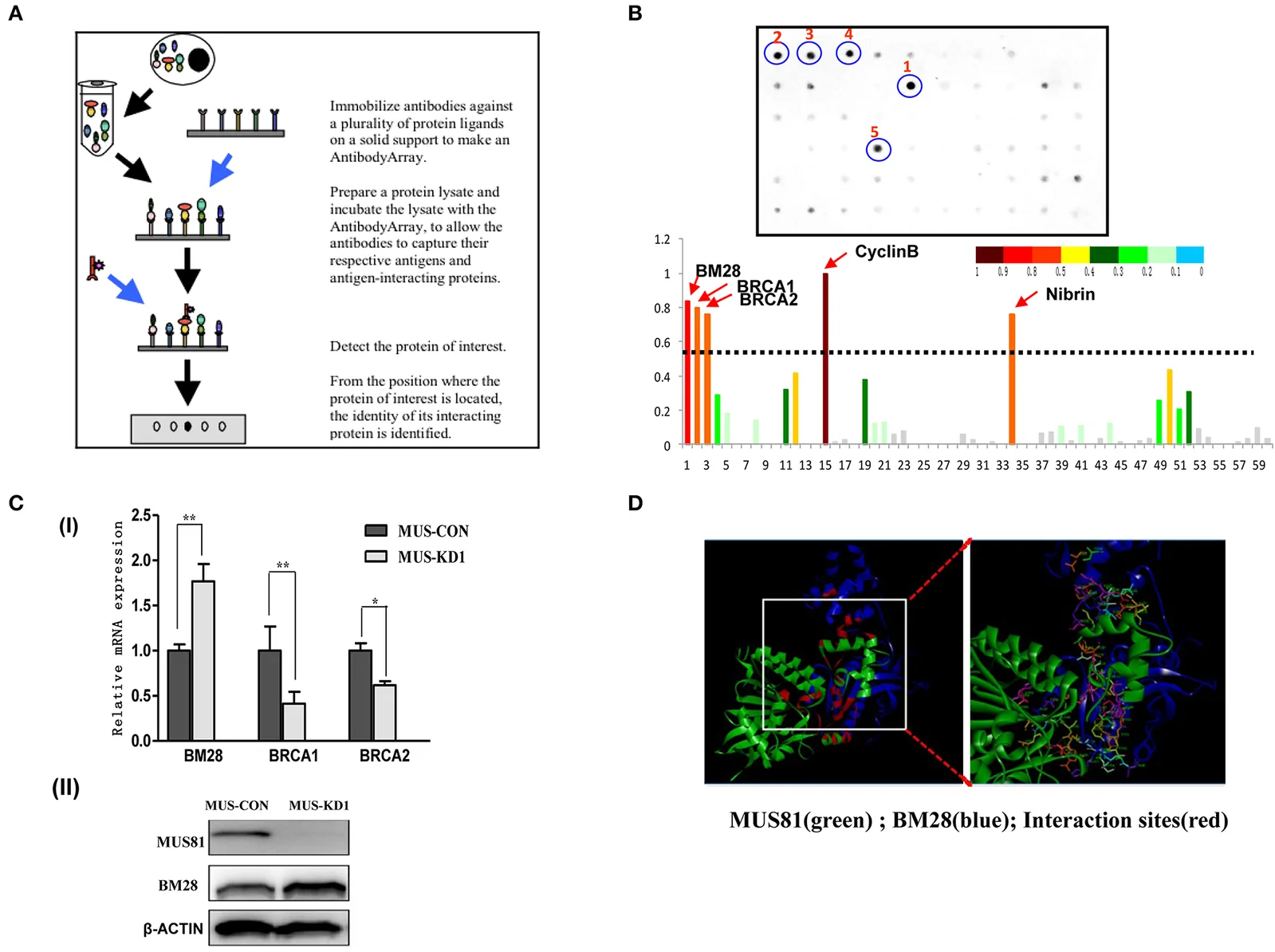
Interactions between MUS81 and proteins involved in cell cycle progression. **(A)** The workflow of the Cell Cycle AntibodyArray™. **(B)** The results of the Cell Cycle AntibodyArray™ assay. Proteins captured on the array were detected by immunoblotting (the positions of 1–5 are CyclinB, BM28, BRCA1, BRCA2, and Nibrin proteins, respectively), and the number of target proteins was calculated by gray scanning. **(C)** The relationships between MUS81 and other proteins were confirmed by western blot or qPCR. (I) The levels of *MUS81* and *BRCA1, BRCA2* mRNA were assayed by qPCR. (II) The levels of MUS81 and BM28 protein expression were analyzed by western blot. Data are presented as the mean ± SD of three independent experiments (^*^P < 0.05, ^**^P < 0.01). **(D)** The potential interaction of MUS81 and BM28 was analyzed by *Discover Studio*.

As the above description, GO-analysis revealed several pathways associated with MUS81 modulation. Importantly, MUS81 was also found to be involved in cell cycle progression, which could contribute to SOC progression. However, it is unclear if MUS81 is actually involved in the SOC cell cycle process; therefore, we further explored the specific protein binding partners of MUS81 using a Cell Cycle Antibody Array™ to screen potential protein ligands. The brief principle of the Cell Cycle Antibody Array is shown in Figure 3A. Consistent with the results of GO-analysis, the protein array showed that MUS81 could interact with some cell cycle molecules including BRCA1, BRCA2, BM28, Cyclin B, and Nibrin, which are involved in DNA-dependent damage repair and the cell cycle (Figure 3B).

To validate the results of the protein array, we identified the relations between the expression of MUS81 and cell cycle-related proteins using qPCR and Western blot. As indicated in Figure 3C, MUS81 knockdown induced an upregulation of BM28, whereas it resulted in a downregulation of BRCA1 and BRCA2. We further used the *Discover Studio* platform to mimic potential interactions between MUS81 and BM28 by modeling the binding of BM28 with expected interactions with crucial amino acids in the active site of MUS81. The stability of the best docked interface of these molecules was evaluated by determining the hydrogen bonding interactions of MUS81 and BM28. These were consistent with the GOLD fitness scores in the protein array that had a better binding affinity between MUS81 and BM28. As shown in Figure 3D, the data also revealed the binding sites of crucial amino acids (red) involved in hydrogen bond formation.

### The Combination of MUS81 and RAD51 Suppression Impacts the Sensitivity of SOC Cells to PARP Inhibitor

**Figure 4:**
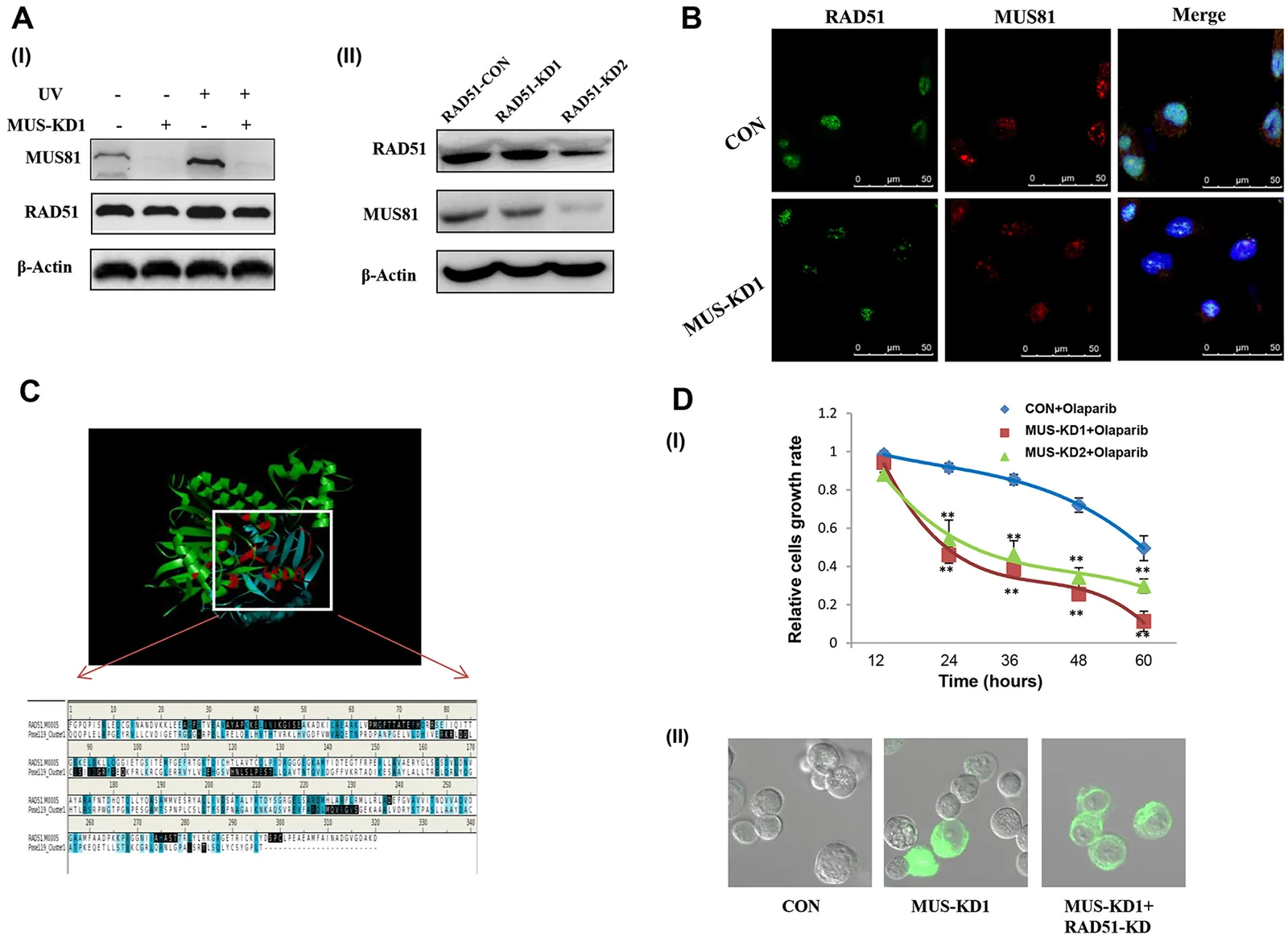
The interaction between MUS81 and RAD51 influenced SOC (SKOV3 and HO8910) cells' sensitivity to DNA-damaging agents. **(A)** The association of MUS81 and RAD51 expression was evaluated. (I) Silencing MUS81 resulted in the decreased expression of RAD51, and both MUS81 and RAD51 were upregulated after UV exposure. (II) RAD51 knockdown was accompanied by downregulation of MUS81. **(B)** The co-localization of MUS81 and RAD51 was observed by immunofluorescence detection. MUS81 was co-localized with RAD51 in the nuclei. **(C)** The potential interaction between MUS81 and RAD51 was analyzed by *Discover Studio*. **(D)** MUS81 suppression was involved in SOC cells sensitivity to DNA damage. (I) MUS81 knockdown enhanced SOC cells sensitivity to the DNA-damaging agent Olaparib (^**^P < 0.01). (II) Simultaneous suppression of MUS81 and RAD51 in the transduced SOC cells was associated with around 70% of cells undergoing apoptosis, whereas 30–40% of MUS-KD cells and only 5% of MUS-CON had evidence of apoptosis. Apoptosis was detected by evaluating the ability of cells to bind lactadherin, which interacts with phosphatidylserine exposed during apoptosis.

HR plays an important role in genome stability and DNA damage repair, and HR activity is affected by many proteins such as BRCA1, BRCA2, and RAD51 (14). Our data showed that RAD51 expression was downregulated with the inhibition of MUS81 (Figure 4A), and the expression of these two proteins was increased simultaneously after UV exposure. To further identify the association between MUS81 and RAD51, we established RAD51 knockdown cells via lentiviral-mediated RNAi. In accord, RAD51 expression in the transduced cells was significantly reduced, which was accompanied by a decrease of MUS81 expression (Figure 4AII). Furthermore, MUS81 colocalized with RAD51 in the nucleus by immunofluorescence staining (Figure 4B). Next, the structure and interaction of these two proteins were mimicked and analyzed using *Discover studio*. The calculation data indicated that RAD51 (green) showed the appropriated interactions with MUS81 (blue) in the primary protein structure (Figure 4C).

We previously revealed the activity of HR after silencing MUS81 in SOC cells, and inhibiting MUS81 expression in SKOV3 cells led to a significant decrease in relative HR activity (19). Olaparib is a poly-ADP ribose polymerase (PARP) inhibitor that impacts cells that lack competent HR repair in the presence of DSBs (20). The combination of HR suppression, by a transgenic technique such as RAD51 RNAi or chemical agent Olaparib, and MUS81 inhibition caused a significant increase in apoptotic death in all SOC cells lines tested relative to either treatment alone. Therefore, MUS81-KD cells were demonstrated to be more sensitive to HR inhibition (Figures 4DI,II).

### Silencing MUS81 Contributes to a Dysfunctional DNA Repair System and CPT Sensitivity

**Figure 5:**
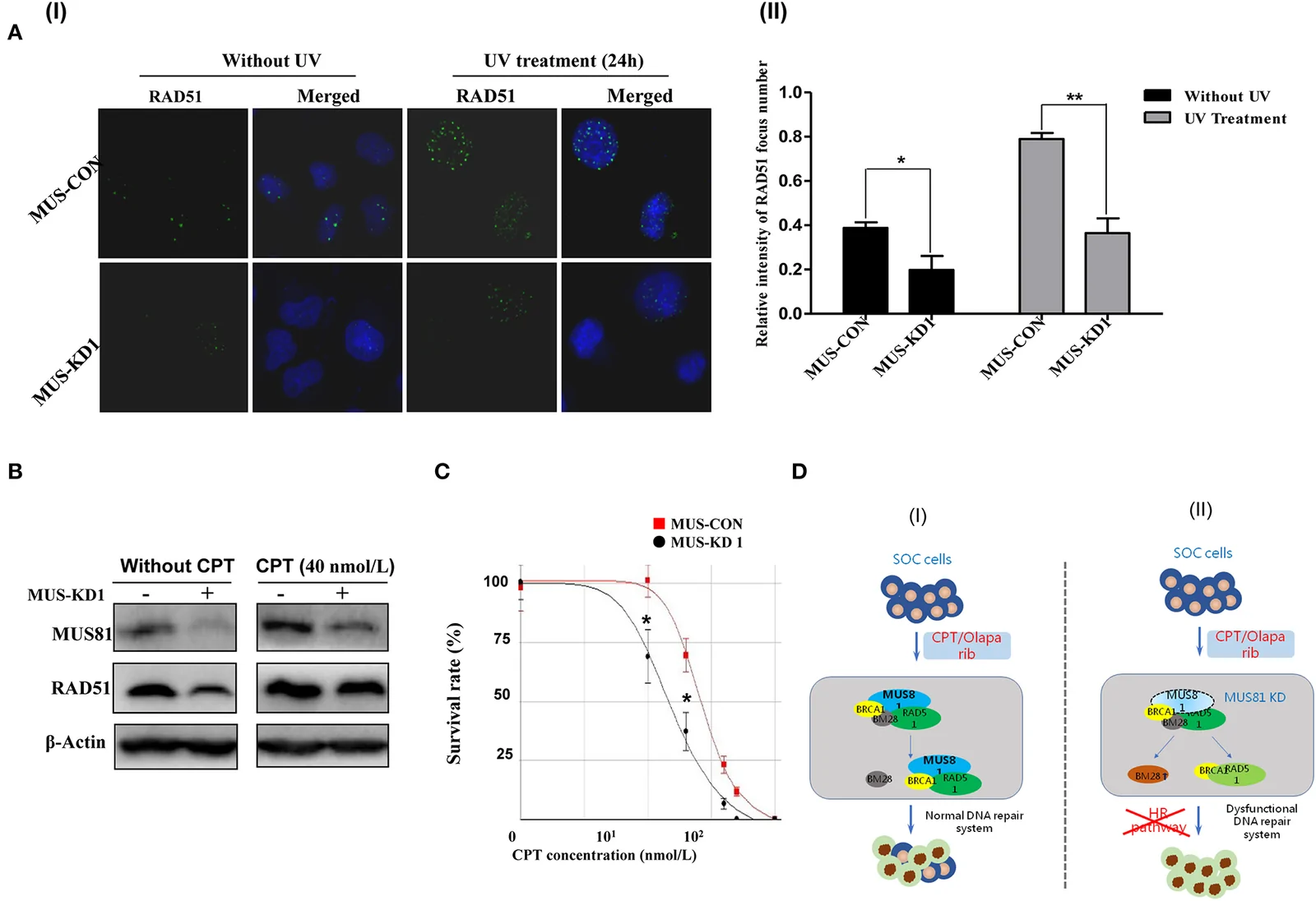
Silencing MUS81 and RAD51 contributes to a dysfunctional DNA repair system. **(A)** The formation of RAD51 foci was inhibited in MUS81 knockdown cells (SKOV3). MUS-KD cells were unable to elicit RAD51 foci in the presence of UV exposure after 24 h, representative images show in (I); Data represent mean ± SD from three independent experiments (II), ^*^P < 0.05, ^**^P < 0.01. **(B)** The levels of RAD51 and MUS81 expression in SKOV3 cell were upregulated significantly after treatment with CPT 4 h. **(C)** MUS81 knockdown increased the sensitivity of SKOV3 cells to CPT. ^*^P < 0.05. **(D)** A schematic diagram of the proposed molecular model of MUS81 intermediates dysfunctional DNA repair systems and drug sensitivity. The dysfunctional DNA repair system or impaired HR pathway by MUS81 inhibition could enhance the effect of therapeutic drugs (CPT/Olaparib) on SOC cells.

To further explore whether MUS-KD cells harbored HR defects, we detected RAD51 foci in the presence of UV-induced DNA DSBs, as it has been reported that the inability of cancer cells to form RAD51 foci could be a surrogate for dysfunctional HR (21, 22). As shown in Figure 5A, when cells were exposed to UV, the expression of MUS81 and RAD51 were consistently increased; furthermore, the formation of RAD51 foci was remarkably decreased in MUS-KD cells compared to the control cells. These data suggested that MUS81 knockdown blocked RAD51 foci formation in the presence of DSBs. CPT is a chemotherapeutic agent that exhibits anti-cancer activity due to its inhibition of DNA topoisomerase I, inducing DNA damage and DSB formation (23). As is shown in Figure 5B, RAD51 expression was decreased in MUS-KD cells, even in those treated with CPT. However, MUS81 and RAD51 protein expression were increased after treatment with CPT 4 h. Coincidently, further data showed that MUS-KD cells were more sensitive to CPT than the control cells according to the proliferation rates of transduced cells with CPT treatment at varying concentrations (Figure 5C). *In vitro* experiments performed by the primary culture cell SOC1 also have shown that MUS81 downregulation had an important impact on the viability and proliferation of the transduced cells with the different concentration and time of CPT treatment, respectively, compared with the untreated controls (Supplementary Figures 2A,B, respectively). As was demonstrated above, both the reproduction and growth of MUS-KD cells treated by CPT were decreased significantly compared to the controls. On the whole, given the collaboration between the abnormal MUS81/RAD51 level and the dysfunctional HR, a schematic diagram of the proposed functional model of MUS81 and the molecules involved in DNA damage and repair is presented in Figure 5D.

### Silencing MUS81 Impacts the Anti-cancer Activity of CPT *in vivo*

**Figure 6:**
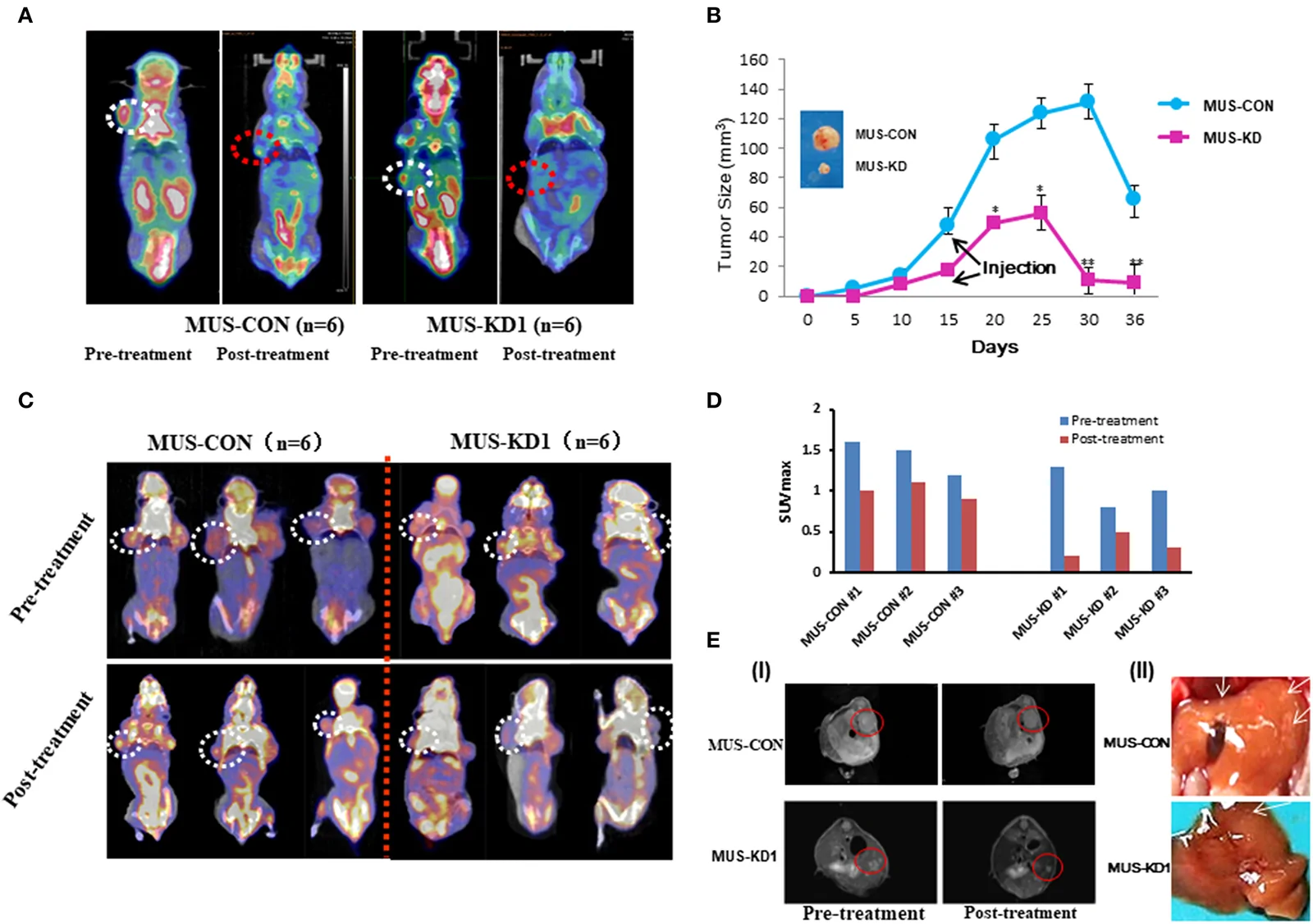
Impact of MUS81 suppression on the anti-tumor activity of CPT. **(A)** Representative whole-body coronal micro-PET CT images of BALB/c mice bearing SOC 1 h after intravenous injection of ^18^F-FDG (1.85 MBq/mouse) on day 14 after inoculation or day 7 after treatment initiation. Tumors are indicated in the circles. **(B)** Tumor volumes were measured in the CON and MUS-KD groups (*n* = 6, respectively); line graph shows average tumor size in mice. **(C)** Evidence of increased chemotherapy sensitivity of tumors with reduced MUS81 levels using the micro-PET CT and the imaging reagent FLT. The 20-day tumor bearing mice in two groups (*n* = 6, respectively) were injected CPT (10 mg/kg) on d 3, 6, and 9; representative whole-body coronal micro-PET CT images of mice with SOC 1.5 h after intravenous injection of ^18^F-FLT (1.85 MBq/mouse) on day 12. **(D)** Quantitative ROI analysis of tumor uptake from ^18^F-FLT micro-PET CT. **(E)** Inhibition of MUS81 reduced tumor volumes and liver metastasis. Transduced SKOV3 cells were injected intraperitoneally to make a BALB/c mice SOC tumorigenesis model. Tumors were scanned by Micro-MR imaging (I). Representatives of liver metastasis of tumors in MUS-KD group and CON group (arrow represents the site of metastasis) (II).

To evaluate the impact of MUS81 on the anti-cancer activity of CPT *in vivo*, a tumorigenesis model using transduced SKOV3 cells was established via subcutaneous injection. Molecular imaging by micro-PET CT with an FDG probe was used to evaluate the biological activity and metabolic status of tumor cells. Representative MUS-KD and MUS-CON cases with or without CPT treatment were made by intravenous injection of CPT (10 mg/kg, twice per week). This treatment led to delayed tumor growth, especially in the MUS-KD group. Additionally, there were significant differences in tumor size between the MUS-KD and MUS-CON group 15–36 days after inoculation (Figures 6A,B). Furthermore, tumor growth was remarkably inhibited after CPT treatment in the MUS-KD group.

3′-deoxy-3′-^18^F-fluorothymidine (^18^F-FLT) is another widely used PET molecular imaging probe that has presented superiority in predicting early therapeutic responses due to its unique cellular uptake mechanism that involves the DNA synthesis pathway via thymidine kinase-1. To verify the impact of CPT on SKOV3 cell proliferation *in vivo*, the MUS-KD and MUS-CON tumor-bearing mice were injected and imaged with ^18^F-FLT. As is shown in Figure 6C, static micro-PET CT scan 1 h after injection was acquired on day 15 (initiated from treatment). Compared with the MUS-CON group, the SUV_max_ of the MUS-KD group was significantly decreased, especially after CPT treatment (Figure 6D). Additionally, the effect of MUS-KD on enhancing the sensitivity to CPT was also confirmed by Micro-MR imaging. Tumor volumes in the MUS-KD group (*n* = 6) were significantly smaller than those of the MUS-CON group in mice treated with CPT, and the liver metastasis of tumor was reduced simultaneously (Figure 6E).

## Discussion

Primary surgical cytoreduction followed by chemotherapy remains the standard-of-care for SOC; however, there remains the problem of genomic adaptability in SOC cells with chemotherapeutic drugs leading to their continual survival. Further, SOC is a disease with synthetic lethality, which is a genetic concept based on disrupting two biological pathways leading to cell death. Recent studies have indicated that the majority of SOC cases are characterized by disruptions in genes involved in the HR pathway of DNA repair (24, 25).

MUS81 is involved in DNA repair, gene replication and cell growth, which indicates a complex dynamic role in subclonal evolution (26, 27). MUS81 abnormal expression was suggested to be associated with clinical outcome (*Well* vs. *Poor, P* = 0.029), although we observed it in two cohorts of 22 SOC clinical samples. Similarly, the expression of MUS81 has some correlation with the clinical stage of SOC. Our data suggested that elevated MUS81 expression was associated with the progression of SOC, especially our findings in the primary cultural cells. Correspondingly, deficiency of MUS81 expression could increase the genomic instability of SOC cells, which in turn causes cancer cells to experience higher levels of “replicative stress;” even replication fork progression in BRCA2-deficient cells requires MUS81 (28). MUS81 provides a mechanism for replication stress tolerance, which can be exploited therapeutically, for the reason that the dysregulated division of cancer cells causes DNA damage.

MUS81 alterations are involved in dysfunction of the DNA repair system. Interestingly, we found that MUS81 participated in the genomic instability that is characteristic of SOC progression. RAPD array also confirmed genome instability as band shifts, missing bands and/or band intensity changes in MUS-KD cells. MUS81 cleaves late replication intermediates during early mitosis to trigger DNA repair and promotes DNA synthesis (29). Thus, inhibition of MUS81 leads to the accumulation of DNA damage in G1 stage cells. BM28 (MCM2) is an important molecule in the highly conserved MCM complex, which participates in the formation of a hexametric protein complex that initiates eukaryotic DNA replication. Strikingly, our previous work revealed that BM28 is closely correlated with MUS81(19). Here, we verified that MUS81 has physical interaction with BM28 and promotes mitotic DNA synthesis in the G1 stage of the cell cycle, and the RAD51 interacting domain causes excessive RAD51 binding to loci and impairs gene expression. These data suggest that MUS81 collaborates with RAD51 in stabilizing stalled replication forks and contributes to genomic instability after DNA damage (30). *Discover Studio* was used to mimic potential interactions between MUS81 and RAD51 by binding RAD51 molecules, which showed the expected interactions with the crucial amino acids present in the active site of MUS81. Furthermore, MUS81 as a DNA endonuclease involved in HR repair is also involved in the modification of many cellular proteins by SUMO2/3. Hu et al. (13) demonstrated that MUS81 mutants displayed compromised DNA damage responses after exposure to metal toxins.

Our previous experiments indicated that inhibiting MUS81 expression led to a significant decrease in relative HR activity (19). A defect in the HR pathway results in hypersensitivity to DNA damage, which is due to impaired MUS81-mediated processing of replication forks in part (31, 32). Here, we performed experiments to identify the mechanisms involved in the genome instability regulated by MUS81 and explored its relationship with outcomes of clinical treatment. Using transcriptional profile analysis and interaction protein screening, we found that MUS81 interacted with cell cycle-related proteins including BRCA1, BRCA2, RAD52, BM28, Cyclin B, and Nibrin, which are involved in DNA repair and cell cycle progression (Figure 3B). Ghamrasni et al. reported that combinatorial loss of RAD54 and MUS81 results in hypersensitivity to DNA-damaging agents; defects in both HR and non-homologous end joining (NHEJ) repair pathways reduced fertility (33). To date, stabilization of stalled DNA replication forks was a recently identified PARPi-resistance mechanism that promotes genomic stability in BRCA1/2-deficient cancers (34). Our data uncovered that downregulation of MUS81 brought a higher sensitivity to the chemotherapy drugs CPT and Olaparib; therefore, we proposed a novel anti-cancer strategy that involves inhibiting MUS81. Furthermore, the combination of MUS81 and RAD51 inhibition contributes to a dysfunctional DNA repair system; it has also been reported that MUS81 triggers Rad51-independent homology-directed repair of collapsed replication forks (35, 36). Moreover, the MUS-KD and MUS-CON ovarian tumor-bearing mice were injected and imaged with ^18^F-FLT and FDG. We found that MUS81 suppression attenuated the tumorigenicity of transduced SOC cells. Additionally, the effects of silencing MUS81 on enhancing CPT sensitivity and inhibiting tumor metastasis were also confirmed by micro-MRI. Recent studies proposed the identification of new targets to predict treatment responses to DNA replication stress (37).

In summary, this study demonstrated that MUS81 might participate in the progression of SOC associated with dysfunctional DNA repair and revealed that MUS81 silencing could promote chemotherapy sensitivity in SOC. Recently, Anita Palma and Aiello FA reported that CK2-dependent phosphorylation to MUS81 at Ser87 is important in mitosis and replication stress (38, 39). In future work, we will try to clarify the specific mechanism of MUS81 in ovarian cancer.

## Data Availability Statement

All datasets generated for this study are included in the article/Supplementary Material.

## Ethics Statement

Specimens isolated from SOC tissues (from Tissue Bank, Fudan University Shanghai Cancer Center) were used under a protocol approved by the Ethics Committee of Shanghai Cancer Center, Fudan University (Certification No. 050432-4-1212B). The animal study was approved by the Institutional Animal Care and Use Committee of Shanghai Medical College, Fudan University (LASFDI-20140187).

## Author Contributions

LG and RL are corresponding authors and organized the study. RL and SX performed most of the experiments. RL and MS performed the bioinformatics analyses. YW, HZhe, HZha, WS, MD, AZ, MC, MZ, and XX helped with various experiments and statistical analyses. RL and SX wrote the manuscript. All authors read and approved the final manuscript.

### Conflict of Interest

The authors declare that the research was conducted in the absence of any commercial or financial relationships that could be construed as a potential conflict of interest.

## Correction note

A correction has been made to this article. Details can be found at: 10.3389/fonc.2026.1916333.

## Supplementary Material

Supplementary Figure 1 High MUS81expression is associated with poor prognosis in serous ovarian cancer patients. **(A)** Kaplan-Meier curves for patients with serous ovarian cancer based on MUS81 expression. Progression-free survival (PFS) curves showed that high MUS81 expression remained a poor clinical outcome (*n* = 22, *P* = 0.0118). **(B)** IHC was performed to assess MUS81 expression in different prognosis SOC patients.

Supplementary Figure 2 Downregulation of MUS81 in the primary SOC1 cells enhanced the sensitivity to CPT. Compared with the control group (MUS-CON), the primary SOC1 cells with knock-down of MUS81 (MUS-KD) were more sensitive to CPT. **(A)** Cells were treated with serial dilutions of CPT ranging from 0 to 3.2 μM for 72 h. **(B)** Cells were treated with CPT (0.8 μM) for 24–96 h.

Supplementary Table 1 Patient and tumor characteristics.

Supplementary Table 2 Target sequences of RNAi and sequences of qRT-PCR primer.

Supplementary Table 3 The relationship between MUS81 levels and clinical outcome in SOC patients.
