# Correction: MUS81 participates in the progression of serous ovarian cancer associated with dysfunctional DNA repair system

**DOI:** 10.3389/fonc.2026.1916333

**Published:** 2026-09-03

**Authors:** Renquan Lu, Suhong Xie, Yanchun Wang, Hui Zheng, Hongqin Zhang, Minjie Deng, Weizhong Shi, Ailing Zhong, Miaomiao Chen, Meiqin Zhang, Xiaofeng Xu, Masood A. Shammas, Lin Guo

**Affiliations:** 1 Department of Clinical Laboratory, Fudan University Shanghai Cancer Center, Shanghai, China; 2 Department of Oncology, Shanghai Medical College, Fudan University, Shanghai, China; 3 Department of Clinical Laboratory, Shanghai Proton and Heavy Ion Center, Shanghai, China; 4 Department of Gynecological Oncology, Fudan University Shanghai Cancer Center, Shanghai, China; 5 Department of Medical Oncology, Dana Farber (Harvard) Cancer Institute, Boston, MA, United States

**Keywords:** ovarian cancer, MUS81, DNA damage, homologous recombination (HR), chemotherapy

In the original article, there was a mistake in **Figure 4** as published. Due to an oversight during the data organization process, two fluorescence images in panel D (II) were inadvertently taken from a parallel study on esophageal cancer and mistakenly used in place of the corresponding ovarian cancer experimental results. Upon verifying the original fluorescence image data, the corrected **Figure 4** and its caption appear below.

**Figure 4:**
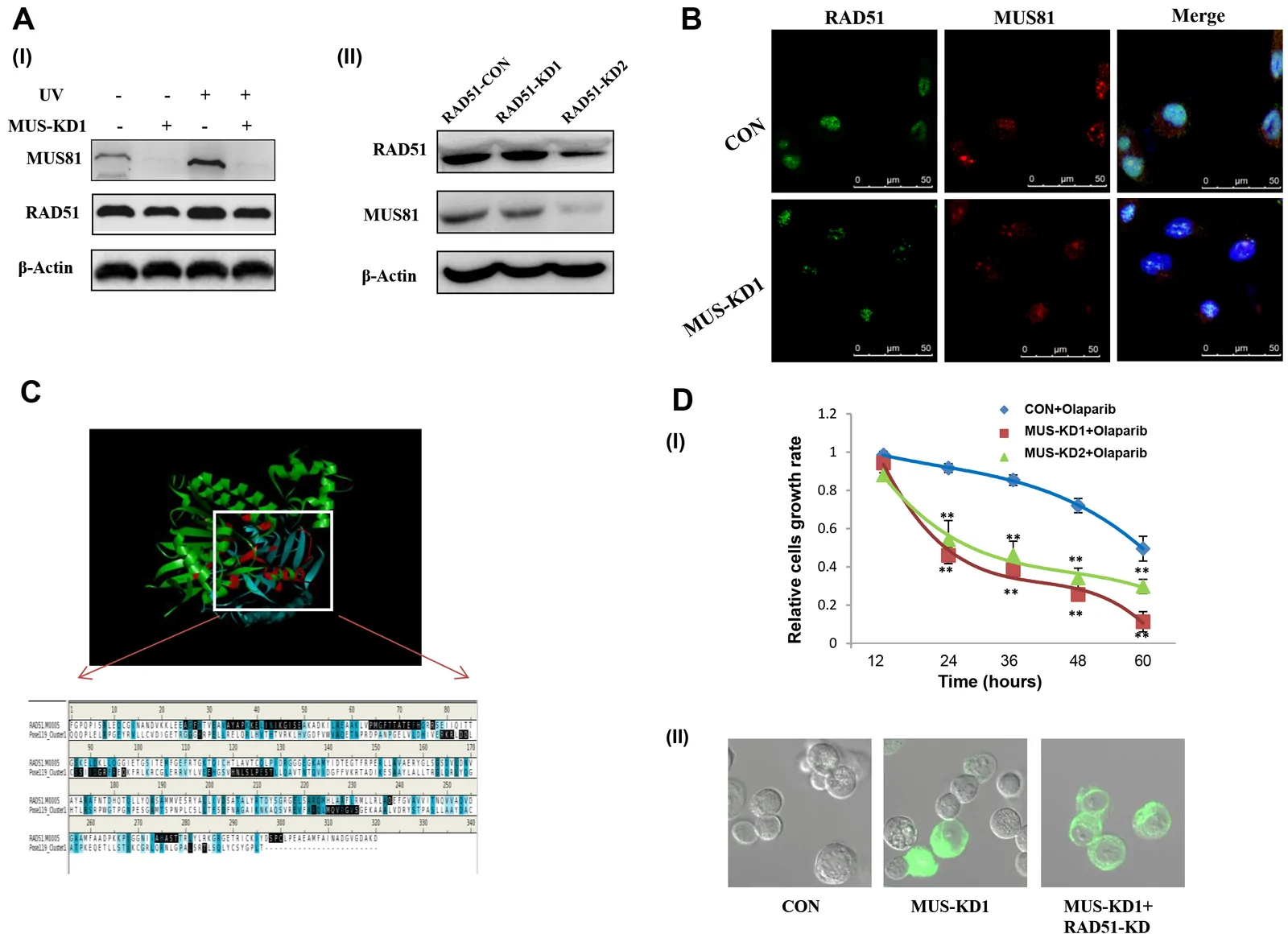
The interaction between MUS81 and RAD51 influenced SOC (SKOV3 and HO8910) cells’ sensitivity to DNA-damaging agents. **(A)** The association of MUS81 and RAD51 expression was evaluated. (I) Silencing MUS81 resulted in the decreased expression of RAD51, and both MUS81 and RAD51 were upregulated after UV exposure. (II) RAD51 knockdown was accompanied by downregulation of MUS81. **(B)** The co-localization of MUS81 and RAD51 was observed by immunofluorescence detection. MUS81 was co-localized with RAD51 in the nuclei. **(C)** The potential interaction between MUS81 and RAD51 was analyzed by Discover Studio. **(D)** MUS81 suppression was involved in SOC cells sensitivity to DNA damage. (I) MUS81 knockdown enhanced SOC cells sensitivity to the DNA-damaging agent Olaparib (**P < 0.01). (II) Simultaneous suppression of MUS81 and RAD51 in the transduced SOC cells was associated with around 70% of cells undergoing apoptosis, whereas 30–40% of MUS-KD cells and only 5% of MUS-CON had evidence of apoptosis. Apoptosis was detected by evaluating the ability of cells to bind FITC-labeled lactadherin, which interacts with phosphatidylserine exposed during apoptosis.

The original article has been updated.

