# Supplementary figures and images for: MUS81 Participates in the Progression of Serous Ovarian Cancer Associated With Dysfunctional DNA Repair System
Source: Front Oncol. 2019 Nov 15;9:1189. doi: 10.3389/fonc.2019.01189 (PMC6873896; doi:10.3389/fonc.2019.01189)

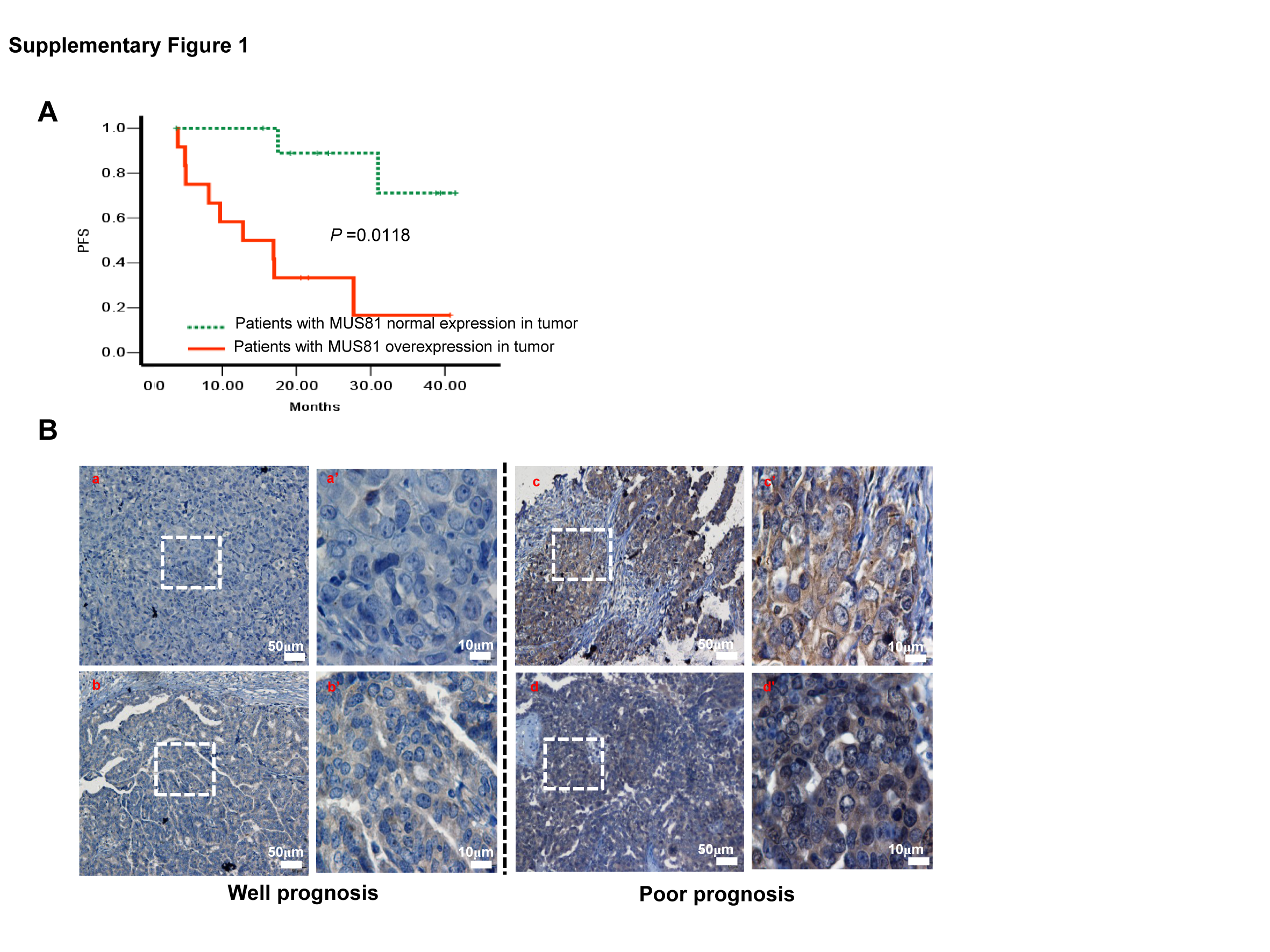

Supplement: Supplementary Figure 1 — High MUS81expression is associated with poor prognosis in serous ovarian cancer patients. (A) Kaplan-Meier curves for patients with serous ovarian cancer based on MUS81 expression. Progression-free survival (PFS) curves showed that high MUS81 expression remained a poor clinical outcome (n = 22, P = 0.0118). (B) IHC was performed to assess MUS81 expression in different prognosis SOC patients. [file Image_1.TIF]

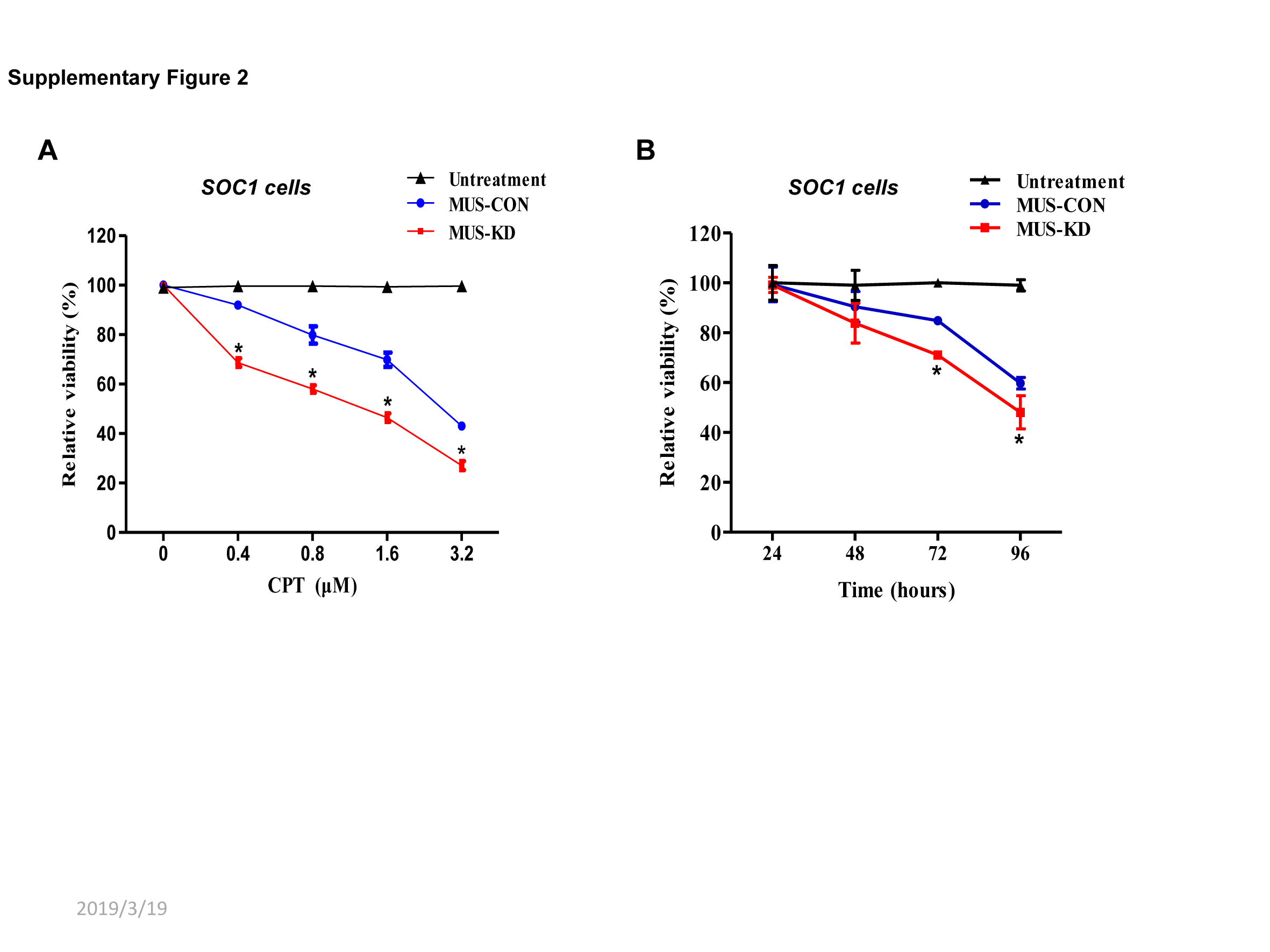

Supplement: Supplementary Figure 2 — Downregulation of MUS81 in the primary SOC1 cells enhanced the sensitivity to CPT. Compared with the control group (MUS-CON), the primary SOC1 cells with knock-down of MUS81 (MUS-KD) were more sensitive to CPT. (A) Cells were treated with serial dilutions of CPT ranging from 0 to 3.2 μM for 72 h. (B) Cells were treated with CPT (0.8 μM) for 24–96 h. [file Image_2.TIF]
