# Supplementary material for: MUS81 Participates in the Progression of Serous Ovarian Cancer Associated With Dysfunctional DNA Repair System
Source: Front Oncol. 2019 Nov 15;9:1189. doi: 10.3389/fonc.2019.01189 (PMC6873896; doi:10.3389/fonc.2019.01189)
Supplement: Supplementary Table 1 — Patient and tumor characteristics. [file Table_1.DOC]

**Supplemental Table 1.**

Patient and tumor characteristics

| Feature | Case |
| --- | --- |
| Age (years) |  |
| ≥55 | 15(68.2%) |
| < 55 | 7(31.8%) |
| Clinical stage |  |
| Early stage(I-II) | 2(9.1%) |
| Advanced stage(III- IV) | 20(90.9%) |
| Peritoneal or omentum metastasis |  |
| Positive (+) | 19(95.5%) |
| Negtive (-) | 3(4.5%) |
| The volume of ascites  ≥1000ml | 14(63.6%) |
| <1000ml | 8(36.4%) |
| Tumor size(cm) |  |
| ≥5 | 15(68.2%) |
| <5 | 7(31.8%) |

|  |  |
| --- | --- |
