# Supplementary material for: MUS81 Participates in the Progression of Serous Ovarian Cancer Associated With Dysfunctional DNA Repair System
Source: Front Oncol. 2019 Nov 15;9:1189. doi: 10.3389/fonc.2019.01189 (PMC6873896; doi:10.3389/fonc.2019.01189)
Supplement: Supplementary Table 2 — Target sequences of RNAi and sequences of qRT-PCR primer. [file Table_2.DOCX]

**Supplemental Table 2.**

Target sequences of RNAi and sequences of qRT-PCR primer

| Primers | Sequences |
| --- | --- |
| RAD51-KD1  RAD51-KD2  CON (scrambled sequence)  MUS81 qRT-PCR forward | 5′-GCTGAAGCTATGTTCGCCATT-3′  5′-CGGTCAGAGATCATACAGATT-3′  5′-CCTAAGGTTAAGTCGCCCTCG-3′  5′-CTGAAGCGCTGTGGTCTG-3′ |
| MUS81 qRT-PCR reverse | 5′-AGTGTTGGTGACAGCCTG-3 |
| BM28 qRT-PCR forward | 5′- CGGAATCATCGGAATCCTTCA-3′ |
| BM28 qRT-PCR reverse | 5′-TCCTCATCATCCAGAGCCAGTC-3′ |
| BRCA1 qRT-PCR forward | 5′-TTGTTACAAATCACCCCTCAAGG-3′ |
| BRCA1 qRT-PCR reverse | 5′-CCCTGATACTTTTCTGGATGCC-3′ |
| BRCA2 qRT-PCR forward | 5′-ACAAGCAACCCAAGTGTCAAT-3′ |
| BRCA2 qRT-PCR reverse | 5′-TGAAGCTACCTCCAAAACTGTG-3′ |
| β-actin qRT-PCR forward | 5′- AAGGTGACAGCAGTCGGTT-3′ |
| β-actin qRT-PCR reverse | 5′- TGTGTGGACTTGGGAGAGG-3′ |
