# Supplementary material for: MUS81 Participates in the Progression of Serous Ovarian Cancer Associated With Dysfunctional DNA Repair System
Source: Front Oncol. 2019 Nov 15;9:1189. doi: 10.3389/fonc.2019.01189 (PMC6873896; doi:10.3389/fonc.2019.01189)
Supplement: Supplementary Table 3 — The relationship between MUS81 levels and clinical outcome in SOC patients. [file Table_3.DOC]

**Supplemental Table 3.**

The relationship between MUS81 levels and clinical outcome in SOC patients

| MUS81 expression | Poor | | Well | *P*-value |
| --- | --- | --- | --- | --- |
| Negative | | 2 | 5 | 0.029 |
| Weak | | 2 | 5 |  |
| Strong | | 7 | 1 |  |
